# Diaqua­bis­{2-hy­droxy-5-[(pyridin-2-yl)methyl­idene­amino]­benzoato-κ^2^ *N*,*N*′}nickel(II) dihydrate

**DOI:** 10.1107/S1600536810046118

**Published:** 2010-11-13

**Authors:** Meiqin Zha, Xing Li, Yue Bing, Zhengbing Luo

**Affiliations:** a Faculty of Materials Science and Chemical Engineering, Ningbo University, Ningbo, Zhejiang 315211, People’s Republic of China

## Abstract

In the title complex, [Ni(C_13_H_9_N_2_O_3_)_2_(H_2_O)_2_]·2H_2_O, the Ni^II^ atom, located on a twofold rotation axis, is in a distorted octa­hedral geometry, defined by four N atoms from two 2-hy­droxy-5-[(pyridin-2-yl)methyl­idene­amino]­benzoate ligands and two O atoms from two water mol­ecules. In the crystal, inter­molecular O—H⋯O hydrogen bonds link the complex mol­ecules and uncoordinated water mol­ecules into a three-dimensional network. Intra­molecular O—H⋯O hydrogen bonds are present between the hy­droxy and carboxyl­ate groups.

## Related literature

For the biological activity of Schiff base compounds, see: Ali *et al.* (2002 ▶); Cukurovali *et al.* (2002 ▶); Tarafder *et al.* (2002 ▶).
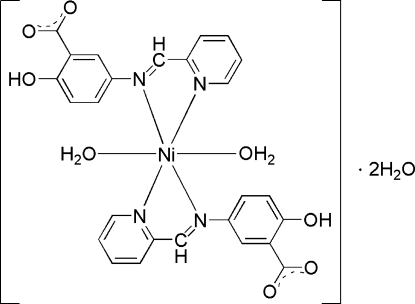


## Experimental

### 

#### Crystal data

[Ni(C_13_H_9_N_2_O_3_)_2_(H_2_O)_2_]·2H_2_O
*M* *_r_* = 613.22
Orthorhombic, 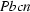

*a* = 15.7628 (6) Å
*b* = 10.5672 (3) Å
*c* = 15.6178 (6) Å
*V* = 2601.44 (16) Å^3^
*Z* = 4
Mo *K*α radiation
μ = 0.81 mm^−1^
*T* = 173 K
0.41 × 0.34 × 0.12 mm

#### Data collection

Bruker APEXII CCD diffractometer
Absorption correction: multi-scan (*SADABS*; Sheldrick, 1996 ▶) *T*_min_ = 0.725, *T*_max_ = 0.907
14975 measured reflections
2294 independent reflections
1599 reflections with *I* > 2σ(*I*)
*R*_int_ = 0.044

#### Refinement

*R*[*F* ^2^ > 2σ(*F* ^2^)] = 0.030
*wR*(*F* ^2^) = 0.074
*S* = 0.89
2294 reflections
202 parameters
4 restraints
H atoms treated by a mixture of independent and constrained refinement
Δρ_max_ = 0.20 e Å^−3^
Δρ_min_ = −0.41 e Å^−3^

### 

Data collection: *APEX2* (Bruker, 2007 ▶); cell refinement: *SAINT* (Bruker, 2007 ▶); data reduction: *SAINT*; program(s) used to solve structure: *SHELXS97* (Sheldrick, 2008 ▶); program(s) used to refine structure: *SHELXL97* (Sheldrick, 2008 ▶); molecular graphics: *SHELXTL* (Sheldrick, 2008 ▶); software used to prepare material for publication: *SHELXTL*.

## Supplementary Material

Crystal structure: contains datablocks I, global. DOI: 10.1107/S1600536810046118/hy2372sup1.cif

Structure factors: contains datablocks I. DOI: 10.1107/S1600536810046118/hy2372Isup2.hkl

Additional supplementary materials:  crystallographic information; 3D view; checkCIF report

## Figures and Tables

**Table 1:** Hydrogen-bond geometry (Å, °)

*D*—H⋯*A*	*D*—H	H⋯*A*	*D*⋯*A*	*D*—H⋯*A*
O3—H3*A*⋯O1	0.84	1.72	2.475 (2)	149
O4—H4*B*⋯O2^i^	0.83 (2)	1.81 (2)	2.621 (2)	168 (3)
O4—H4*C*⋯O5	0.84 (2)	1.91 (2)	2.744 (3)	173 (2)
O5—H5*B*⋯O3^ii^	0.82 (2)	2.05 (2)	2.870 (3)	178 (3)
O5—H5*C*⋯O1^iii^	0.84 (2)	1.99 (2)	2.793 (3)	160 (3)

Symmetry codes: (i) 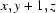; (ii) 
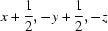
; (iii) 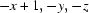.

**Fig. 1.**
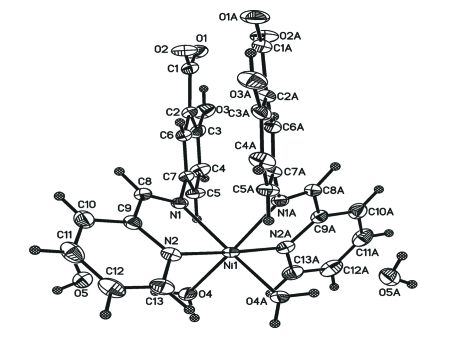
The molecular structure of the title compound. Displacement ellipsoids are drawn at the 30% probability level. [Symmetry code: (A) -x+1, y, -z+1/2.]

**Fig. 2.**
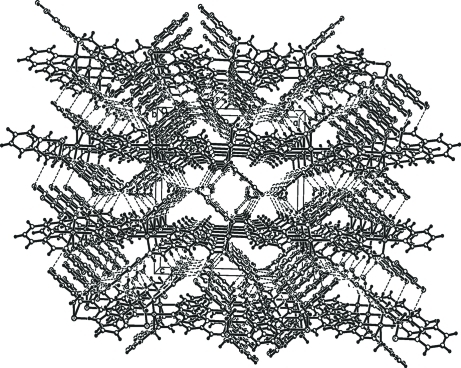
Three-dimensional supramolecular network in the title compound. Dashed lines denote hydrogen bonds.

## supplementary crystallographic information

### Comment

Schiff base compounds have been of great interest for many years. These compounds play an important role in antitumor, antimicrobial and antiviral activities (Ali *et al.*, 2002; Cukurovali *et al.*, 2002; Tarafder *et al.*, 2002). As an extension of the work on the structural characterization of Schiff base compounds, the crystal structure of the title compound is reported here.

The title compound is a mononuclear nickel(II) complex, as shown in Fig. 1. The Ni^II^ atom, lying on a twofold rotation axis, is six-coordinated in a distorted octahedral geometry, defined by four N donors from two Schiff base ligands, two O atoms from two coordinated water molecules. The molecular formula contains two uncoordinated water molecules. The Ni—N bond lengths are 2.0754 (18) and 2.1347 (17) Å, and the Ni—O distance is 2.0380 (17) Å. Intramolecular O—H···O hydrogen bonds between the hydroxy and carboxylate groups are observed (Table 1). In the crystal, intermolecular O—H···O hydrogen bonds link the complex molecules and uncoordinated water molecules into a three-dimensional network (Fig. 2).

### Experimental

5-Aminosalicylic acid (1.53 g, 10 mmol), 2-pyridinecarboxaldehyde (1 ml, 10 mmol) and triethylamine (1 ml, 10 mmol) were mixed in 50 ml ethanol in a round flask. The mixture was refluxed with agitation for 4 h at 323 K to give a yellow precipitate. After filtration and washing the precipitate with ethanol, a pure Schiff base ligand, 5-[(pyridin-2-yl)methyleneamino]-2-hydroxybenzoic acid (yield: 2.02 g, 84%) was obtained.

A mixture of 5-[(pyridin-2-yl)methyleneamino]-2-hydroxybenzoic acid (0.024 g, 0.1 mmol), Ni(CH_3_CO_2_)_2_.2H_2_O (0.025 g, 0.1 mmol) and ethanol (20 ml) was heated at 273 K for 30 min to give a red solution. After evaporating the solution at room temperature for one week, red crystals were obtained (yield: 65%).

### Refinement

H atoms attached to C atoms and O3 were placed in calculated positions and treated using a riding model, with C—H = 0.95 and O—H = 0.84 Å and *U*_iso_(H) = 1.2*U*_eq_(C) or 1.5*U*_eq_(O). H atoms attached to water molecules (O4 and O5) were located in a difference Fourier map and refined isotropically.

### Crystal data

**Table:**

[Ni(C_13_H_9_N_2_O_3_)_2_(H_2_O)_2_]·2H_2_O	*F*(000) = 1272
*M**_r_* = 613.22	*D*_x_ = 1.566 Mg m^−^^3^
Orthorhombic, *P**b**c**n*	Mo *K*α radiation, λ = 0.71073 Å
Hall symbol: -P 2n 2ab	Cell parameters from 14975 reflections
*a* = 15.7628 (6) Å	θ = 3.9–25.0°
*b* = 10.5672 (3) Å	µ = 0.81 mm^−^^1^
*c* = 15.6178 (6) Å	*T* = 173 K
*V* = 2601.44 (16) Å^3^	Platelet, red
*Z* = 4	0.41 × 0.34 × 0.12 mm

### Data collection

**Table:**

Bruker APEXII CCD diffractometer	2294 independent reflections
Radiation source: fine-focus sealed tube	1599 reflections with *I* > 2σ(*I*)
graphite	*R*_int_ = 0.044
φ and ω scans	θ_max_ = 25.0°, θ_min_ = 3.9°
Absorption correction: multi-scan (*SADABS*; Sheldrick, 1996)	*h* = −18→18
*T*_min_ = 0.725, *T*_max_ = 0.907	*k* = −12→12
14975 measured reflections	*l* = −18→13

### Refinement

**Table:**

Refinement on *F*^2^	Primary atom site location: structure-invariant direct methods
Least-squares matrix: full	Secondary atom site location: difference Fourier map
*R*[*F*^2^ > 2σ(*F*^2^)] = 0.030	Hydrogen site location: inferred from neighbouring sites
*wR*(*F*^2^) = 0.074	H atoms treated by a mixture of independent and constrained refinement
*S* = 0.89	*w* = 1/[σ^2^(*F*_o_^2^) + (0.045*P*)^2^] where *P* = (*F*_o_^2^ + 2*F*_c_^2^)/3
2294 reflections	(Δ/σ)_max_ < 0.001
202 parameters	Δρ_max_ = 0.20 e Å^−^^3^
4 restraints	Δρ_min_ = −0.40 e Å^−^^3^

### Fractional atomic coordinates and isotropic or equivalent isotropic displacement parameters (Å^2^)

**Table:**

	*x*	*y*	*z*	*U*_iso_*/*U*_eq_	
Ni1	0.5000	0.40233 (3)	0.2500	0.02879 (13)	
C1	0.42670 (16)	−0.1813 (2)	0.09408 (16)	0.0418 (6)	
C2	0.42072 (13)	−0.04024 (19)	0.08275 (14)	0.0314 (5)	
C3	0.35591 (14)	0.0140 (2)	0.03319 (15)	0.0371 (6)	
C4	0.34858 (15)	0.1436 (2)	0.02799 (15)	0.0422 (6)	
H4A	0.3049	0.1798	−0.0061	0.051*	
C5	0.40373 (14)	0.2213 (2)	0.07156 (14)	0.0376 (5)	
H5A	0.3975	0.3106	0.0685	0.045*	
C6	0.47632 (13)	0.03895 (19)	0.12562 (13)	0.0306 (5)	
H6A	0.5204	0.0032	0.1593	0.037*	
C7	0.46889 (13)	0.16841 (18)	0.12030 (13)	0.0281 (5)	
C8	0.60516 (14)	0.2224 (2)	0.16257 (13)	0.0340 (5)	
H8A	0.6237	0.1479	0.1343	0.041*	
C9	0.66617 (13)	0.3041 (2)	0.20555 (14)	0.0333 (5)	
C10	0.75249 (14)	0.2905 (2)	0.19473 (15)	0.0463 (6)	
H10A	0.7747	0.2214	0.1629	0.056*	
C11	0.80586 (17)	0.3784 (3)	0.23054 (17)	0.0543 (7)	
H11A	0.8656	0.3711	0.2242	0.065*	
C12	0.77113 (16)	0.4765 (3)	0.27551 (17)	0.0529 (7)	
H12A	0.8067	0.5404	0.2989	0.064*	
C13	0.68392 (16)	0.4830 (2)	0.28702 (16)	0.0445 (6)	
H13A	0.6608	0.5500	0.3202	0.053*	
N1	0.52662 (11)	0.25156 (15)	0.16309 (11)	0.0290 (4)	
N2	0.63159 (11)	0.39730 (16)	0.25264 (11)	0.0347 (4)	
O1	0.37216 (11)	−0.24708 (15)	0.05397 (12)	0.0591 (5)	
O2	0.48289 (12)	−0.22398 (14)	0.14171 (12)	0.0561 (5)	
O3	0.29873 (10)	−0.06080 (16)	−0.00716 (11)	0.0531 (5)	
H3A	0.3095	−0.1370	0.0035	0.080*	
O4	0.50698 (13)	0.53118 (16)	0.15316 (12)	0.0501 (5)	
O5	0.62630 (14)	0.48046 (19)	0.02994 (14)	0.0614 (5)	
H4B	0.4935 (15)	0.6066 (17)	0.1536 (18)	0.062 (9)*	
H4C	0.5432 (14)	0.522 (2)	0.1140 (13)	0.055 (9)*	
H5C	0.618 (2)	0.419 (2)	−0.0026 (18)	0.084 (11)*	
H5B	0.6754 (14)	0.505 (3)	0.024 (2)	0.109 (15)*	

### Atomic displacement parameters (Å^2^)

**Table:**

	*U*^11^	*U*^22^	*U*^33^	*U*^12^	*U*^13^	*U*^23^
Ni1	0.0350 (2)	0.0170 (2)	0.0344 (2)	0.000	0.00107 (18)	0.000
C1	0.0476 (16)	0.0267 (13)	0.0512 (15)	−0.0005 (11)	0.0112 (13)	−0.0075 (12)
C2	0.0363 (13)	0.0238 (12)	0.0340 (12)	0.0022 (9)	0.0041 (10)	−0.0050 (10)
C3	0.0412 (14)	0.0343 (14)	0.0359 (12)	0.0005 (10)	0.0030 (11)	−0.0131 (10)
C4	0.0479 (15)	0.0378 (14)	0.0409 (13)	0.0117 (11)	−0.0143 (11)	−0.0048 (11)
C5	0.0493 (14)	0.0233 (12)	0.0402 (13)	0.0051 (10)	−0.0040 (11)	−0.0003 (10)
C6	0.0308 (13)	0.0253 (11)	0.0357 (13)	0.0038 (9)	0.0029 (10)	0.0004 (10)
C7	0.0325 (12)	0.0212 (11)	0.0306 (12)	0.0003 (9)	0.0036 (10)	−0.0048 (9)
C8	0.0411 (15)	0.0244 (12)	0.0364 (13)	0.0011 (10)	0.0046 (11)	0.0001 (10)
C9	0.0329 (13)	0.0308 (13)	0.0362 (13)	−0.0016 (9)	−0.0001 (11)	0.0065 (11)
C10	0.0385 (15)	0.0470 (16)	0.0534 (16)	−0.0012 (11)	0.0037 (12)	0.0096 (13)
C11	0.0358 (14)	0.0617 (19)	0.0654 (19)	−0.0079 (13)	−0.0070 (13)	0.0216 (15)
C12	0.0469 (17)	0.0557 (18)	0.0563 (17)	−0.0195 (14)	−0.0180 (13)	0.0137 (14)
C13	0.0522 (16)	0.0393 (15)	0.0419 (14)	−0.0103 (12)	−0.0117 (12)	0.0048 (12)
N1	0.0346 (11)	0.0211 (9)	0.0313 (10)	0.0009 (7)	−0.0002 (8)	0.0019 (8)
N2	0.0387 (10)	0.0290 (10)	0.0365 (10)	−0.0064 (8)	−0.0045 (9)	0.0039 (9)
O1	0.0604 (12)	0.0302 (10)	0.0869 (13)	−0.0087 (8)	−0.0029 (10)	−0.0191 (9)
O2	0.0731 (13)	0.0220 (9)	0.0731 (12)	0.0054 (8)	−0.0089 (10)	0.0025 (9)
O3	0.0497 (11)	0.0443 (10)	0.0654 (12)	−0.0009 (8)	−0.0160 (9)	−0.0216 (9)
O4	0.0730 (13)	0.0260 (10)	0.0512 (11)	0.0140 (9)	0.0232 (10)	0.0116 (8)
O5	0.0665 (15)	0.0488 (13)	0.0688 (14)	−0.0066 (11)	0.0228 (12)	−0.0147 (11)

### Geometric parameters (Å, °)

**Table:**

Ni1—O4	2.0380 (17)	C8—C9	1.456 (3)
Ni1—N2	2.0754 (18)	C8—H8A	0.9500
Ni1—N1	2.1347 (17)	C9—N2	1.345 (3)
C1—O2	1.241 (3)	C9—C10	1.378 (3)
C1—O1	1.271 (3)	C10—C11	1.372 (3)
C1—C2	1.504 (3)	C10—H10A	0.9500
C2—C6	1.384 (3)	C11—C12	1.367 (4)
C2—C3	1.404 (3)	C11—H11A	0.9500
C3—O3	1.354 (3)	C12—C13	1.388 (3)
C3—C4	1.377 (3)	C12—H12A	0.9500
C4—C5	1.376 (3)	C13—N2	1.337 (3)
C4—H4A	0.9500	C13—H13A	0.9500
C5—C7	1.395 (3)	O3—H3A	0.8400
C5—H5A	0.9500	O4—H4B	0.825 (16)
C6—C7	1.376 (3)	O4—H4C	0.843 (16)
C6—H6A	0.9500	O5—H5C	0.837 (17)
C7—N1	1.431 (3)	O5—H5B	0.823 (18)
C8—N1	1.276 (3)		
			
O4^i^—Ni1—O4	96.16 (11)	C6—C7—C5	119.61 (19)
O4^i^—Ni1—N2	93.23 (8)	C6—C7—N1	121.90 (19)
O4—Ni1—N2	88.74 (7)	C5—C7—N1	118.48 (18)
O4^i^—Ni1—N2^i^	88.74 (7)	N1—C8—C9	119.7 (2)
O4—Ni1—N2^i^	93.23 (8)	N1—C8—H8A	120.2
N2—Ni1—N2^i^	177.06 (9)	C9—C8—H8A	120.2
O4^i^—Ni1—N1	168.85 (7)	N2—C9—C10	122.9 (2)
O4—Ni1—N1	90.93 (7)	N2—C9—C8	114.73 (19)
N2—Ni1—N1	78.29 (7)	C10—C9—C8	122.3 (2)
N2^i^—Ni1—N1	99.48 (7)	C11—C10—C9	119.0 (2)
O4^i^—Ni1—N1^i^	90.93 (7)	C11—C10—H10A	120.5
O4—Ni1—N1^i^	168.85 (7)	C9—C10—H10A	120.5
N2—Ni1—N1^i^	99.48 (6)	C12—C11—C10	118.5 (2)
N2^i^—Ni1—N1^i^	78.29 (7)	C12—C11—H11A	120.7
N1—Ni1—N1^i^	83.45 (9)	C10—C11—H11A	120.7
O2—C1—O1	125.4 (2)	C11—C12—C13	120.0 (2)
O2—C1—C2	118.4 (2)	C11—C12—H12A	120.0
O1—C1—C2	116.2 (2)	C13—C12—H12A	120.0
C6—C2—C3	118.72 (19)	N2—C13—C12	121.7 (2)
C6—C2—C1	120.2 (2)	N2—C13—H13A	119.1
C3—C2—C1	121.0 (2)	C12—C13—H13A	119.1
O3—C3—C4	119.8 (2)	C8—N1—C7	117.79 (18)
O3—C3—C2	120.2 (2)	C8—N1—Ni1	112.01 (14)
C4—C3—C2	120.0 (2)	C7—N1—Ni1	129.08 (13)
C5—C4—C3	120.8 (2)	C13—N2—C9	117.7 (2)
C5—C4—H4A	119.6	C13—N2—Ni1	127.38 (16)
C3—C4—H4A	119.6	C9—N2—Ni1	114.39 (14)
C4—C5—C7	119.7 (2)	C3—O3—H3A	109.5
C4—C5—H5A	120.2	Ni1—O4—H4B	129 (2)
C7—C5—H5A	120.2	Ni1—O4—H4C	120.0 (17)
C7—C6—C2	121.2 (2)	H4B—O4—H4C	107 (3)
C7—C6—H6A	119.4	H5C—O5—H5B	109 (3)
C2—C6—H6A	119.4		

Symmetry codes: (i) −*x*+1, *y*, −*z*+1/2.

### Hydrogen-bond geometry (Å, °)

**Table:**

*D*—H···*A*	*D*—H	H···*A*	*D*···*A*	*D*—H···*A*
O3—H3A···O1	0.84	1.72	2.475 (2)	149
O4—H4B···O2^ii^	0.83 (2)	1.81 (2)	2.621 (2)	168 (3)
O4—H4C···O5	0.84 (2)	1.91 (2)	2.744 (3)	173 (2)
O5—H5B···O3^iii^	0.82 (2)	2.05 (2)	2.870 (3)	178 (3)
O5—H5C···O1^iv^	0.84 (2)	1.99 (2)	2.793 (3)	160 (3)

Symmetry codes: (ii) *x*, *y*+1, *z*; (iii) *x*+1/2, −*y*+1/2, −*z*; (iv) −*x*+1, −*y*, −*z*.
